# Slow Magnetic Relaxation and Modulated Photoluminescent Emission of Coordination Polymer Based on 3-Amino-4-hydroxybenzoate Zn and Co Metal Ions

**DOI:** 10.3390/molecules28041846

**Published:** 2023-02-15

**Authors:** Estitxu Echenique-Errandonea, Sara Rojas, Javier Cepeda, Duane Choquesillo-Lazarte, Antonio Rodríguez-Diéguez, José M. Seco

**Affiliations:** 1 Applied Chemistry Department, Faculty of Chemistry, University of the Basque Country (UPV/EHU), Paseo Manuel de Lardizabal 3, 20018 Donostia-San Sebastián, Spain; 2 Inorganic Chemistry Department, Faculty of Sciences, University of Granada, Avda. Fuentenueva S/N, 18002 Granada, Spain; 3 Laboratory for Crystallographic Studies IACT, CSIC-UGR, Av. Las Palmeras n°4, 18100 Granada, Spain

**Keywords:** metal-organic framework, cobalt-zinc bifunctionality, induced molecular magnetism, photoluminescent properties

## Abstract

As a starting point, a new 3D porous framework with the {[CoL]·0.5DMF·H_2_O}_n_ chemical formula (where L = 3-amino-4-hydroxybenzoate) is described. Its performance as a single molecule magnet was explored. The study of magnetic properties reveals that Co-MOF shows no frequency-fdependant alternating current (*ac*) signals under zero direct current (*dc*) magnetic field, whereas single-molecule magnet behaviour is achieved when Co^II^ ions are diluted in a Zn^II^ based matrix. Interestingly, this strategy renders a bifunctional [Co_x_Zn_1-x_L]_n_ material that is also characterized by a strong photoluminescent emitting capacity.

## 1. Introduction

Multifunctional molecular materials (MMMs) are compounds in which two or more physical properties coexist, compete or cooperate [1]. Therefore, combinations such as conductive/optical, magnetic/optical or conductive/magnetic are possible to study, giving the opportunity of analysing simultaneously the influence of one (or more) physical property present in these materials. Because of the broad spectrum that these materials can cover, several applications can be addressed with MMMs, such as separation and storage, heterogeneous catalysis, drug delivery, sensor devices and magnetic and photoluminescence, among others [2,3,4,5,6,7,8].

In this line, the exploration of new metal–organic frameworks (MOFs) with improved physico-chemical properties are an ongoing prerequisite and aim. MOFs offer the possibility to rationally design the structure of the material in order to shape the desired properties for a particular final application. To that end, the type and properties of the metal ions composing the structure are of great importance. Consequently, the incorporation of different metal ions in the same structure by constructing mixed-metal-organic frameworks (M’MOFs) might be advantageous to prompt a specific application into the material [9,10].

In this work, we have successfully synthesised a novel Co^II^ based MOF and implemented this approach to yield several heterometallic Zn^II^ doped M’MOFs with the aim of systematically studying their magnetic and spectroscopic properties. For this purpose, we took advantage of the isostructurallity shown by our new Co^II^ coordination compound to the previously reported Zn^II^ counterpart [11], which has proven to be a MOF with extraordinary acid–based resistance, and to efficiently separate acetylene from C_2_H_2_/CO_2_ mixtures under ambient conditions showing the highest C_2_H_2_/CO_2_ uptake ratio reported in the bibliography for MOFs to date [12,13,14,15].

On another level, the magnetic behaviour of 3D ions enables their application into molecular magnetism, a field of active research which has contributed to the development of high-density data storage and quantum computation [16]. This magnetic behaviour derives from the slow magnetic relaxation of metal ions with an appropriate coordination shell, in this case Co^II^. At this point, the structural design is thus crucial so that the coordination of organic ligands not only favour the occurrence of magnetic anisotropy but also isolate the spin carriers by imposing weak or null exchange interactions in the crystal building to avoid long-range magnetic ordering phenomena, such as ferromagnetism, antiferromagnetism, and metamagnetism [2,17]. An alternative to avoid those undesired effects is to physically isolate (dilute) the spin carriers into a diamagnetic matrix that preserves isostructural nature, a process called magnetic dilution, in such a way that magnetic exchange interactions would be partially suppressed to give single ion magnetic properties, and hence single-ion magnets (SIMs) [18]. These materials are able to keep the magnetic moment after the exposure to a magnetic field and slowly reorient it as a consequence of the existence of an energy barrier below a blocking temperature. The energy barrier (*U*) for the reversal of spin is dependent on the axial zero-field splitting parameter (*D*) and the total spin of the complex (*S*) [19]. In general, the magnetic behaviour of these 3D ion–based compounds results from the interaction between ligand–field splitting and spin–orbit interaction, both of which can generate large anisotropy (*D*). In particular, Co^II^ complexes are good candidates for the construction of SIMs owing to their large magnetic anisotropy, which is directly influenced by the coordination environment of Co^II^ ions. Trigonal prismatic coordination geometries lead to highly negative values of *D*; therefore, they are very appropriate for the preparation of SIMs. Tetrahedral Co^II^ geometries with s_i_ = 3/2 show a larger spin–orbit coupling constant and hence these complexes can display larger *D* values [20]. In fact, although the highest effective energy barrier exhibited by a Co^II^ complex has been observed in a tetrahedral compound (*U*_eff_ = 118 cm^−1^ at zero direct-current (dc) field), trigonal-prismatic Co^II^ complexes have also shown interesting SMM properties [21]. However, for 3D ion-based SIMs, the SMM behaviour is usually only visible under a small applied external field that suppresses the fast magnetic tunnelling, making that not much mononuclear complexes based on 3D ions show slow relaxation of the magnetization [22].

In our attempt to synthesise a multifunctional mixed MOF, incorporation of Zn^II^ into the network not only plays an important role in inducing magnetic dilution but also gives the possibility to study photoluminescent properties. Group12 metals are well known for their lack of luminescence quenching since they afford no d–d transition and exhibit flexible coordination environments associated with a closed-shell configuration allowing them to be adapted to a wide range of geometries [23,24]. In this sense, ligand-centred (LC) and ligand-to-metal charge transfers (LMCT) may improve their performance in solid samples [25,26]. Solid-state light-emitting MOFs are receiving considerable attention since they can be used as ideal platforms to boost the development of improved devices for applications in light-emitting diodes and optical sensors, among others.

Bearing these ideas in mind, briefly, we have synthesised and characterised a new Co^II^ MOF using 3-amino-4-hydroxybenzoate ligand and studied the magnetic properties. This is an almost unexplored ligand for generating magnetic materials because it has only been studied with lanthanide(III) ions but not in combination with transition metal ions [27]. Regarding the studied magnetic properties, we studied slow magnetic relaxation. In addition, we performed magnetic dilution of the MOF taking advantage of the isostructurality of a Zn^II^ counterpart. Furthermore, we studied homometallic Zn^II^ and Co^II^-Zn^II^ heterometallic materials’ photoluminescent emission properties.

## 2. Results

The solvothermal reaction of the 3-amino-4-hydroxybenzoic acid ligand H_2_L with Co^II^ salt in N,N′-dimethylformamide/water (DMF/H_2_O) mixture yielded a three-dimensional MOF of general formulae {[CoL]·0.5DMF·H_2_O}_n_, namely Co-MOF (see Section 3 for further details).

### 2.1. Crystal Structure Description

**Figure 1:**
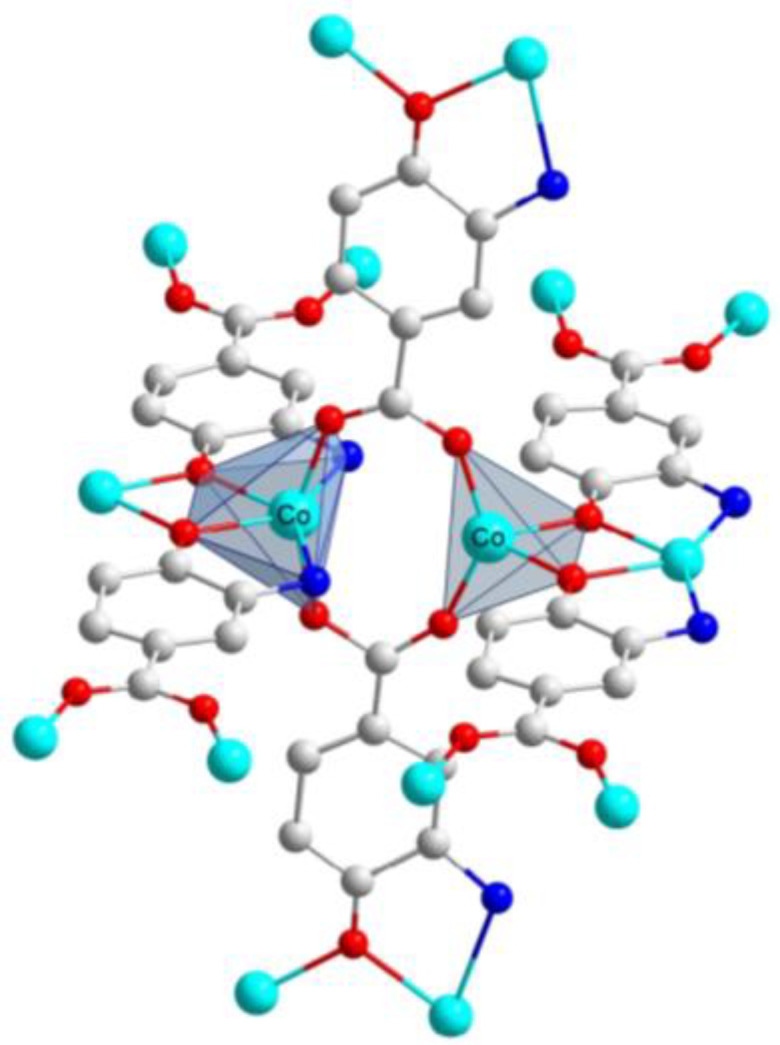
Excerpt of the crystal structure of Co-MOF showing the trigonal prismatic and tetrahedral the coordination polyhedral involved in the disorder of the structure.

The single-crystal X-ray diffraction analysis revealed that Co-MOF crystallizes in the tetragonal *P4_2_* space group as a racemic twin, probably derived from the lower symmetry present in the crystal structure, a fact that generates a systematic disorder in the framework. In fact, although most of the framework possessed high overall symmetry, yielding an asymmetric unit with a unique cobalt ion and a deprotonated ligand, there were indeed two non-equivalent cobalt atoms with distinct coordination environments (Figure 1). One is a trigonal prism SBU, in which Co^2+^ has 6-connected nodes –CoN_2_O_4_ coordination environment– where the metallic centre is linked by two oxygen atoms from different carboxylate groups, two oxygen atoms from different hydroxyl groups, and two nitrogen atoms from two different amino groups. The other coordination environment corresponds to a tetrahedron SBU, in which Co^2+^ displays 4-connected nodes –CoO_4_ coordination environment– where two oxygen atoms from different carboxylate groups and two oxygen atoms from different hydroxyl groups complete the coordination sphere. Continuous shape measurements (CShMs) [28] revealed that the Co(II)-based polyhedra somehow resemble a trigonal prism (TPR) and a tetrahedron (Td), respectively (see Tables S4 and S5), although the first environment is severely distorted owing to the disorder of the crystal structure. On its part, the organic ligand, 3-amino-4-hydroxy benzoato, is also disordered into two equivalent dispositions and shows a tetradentate µ_4_-κO:κO′:κO″,N:κO‴ coordination mode by using the carboxylate, hydroxyl and amino groups to link to both metal centres. Regarding the most representative bond lengths, it can be stated that the distance between cobalt ions if of 3.304 Å; furthermore, Co and nitrogen heteroatom display 2.4091 Å and 3.5469 Å length, Co and O1 1.9367 Å and 2.0802 Å, Co and O2 1.8610 Å and 3.1768 Å, Co and O3 of 1.9145 Å and 3.3833 Å as it is summarized in (Table S3). The linkage of both SBUs by means of the ligands generates a dimeric core that is connected into chains which are further extended in the three directions to give rise to a **pts** topological network with the (4^2^·8^4^) point symbol, according to the topological analysis performed with TOPOS software [29].

**Figure 2:**
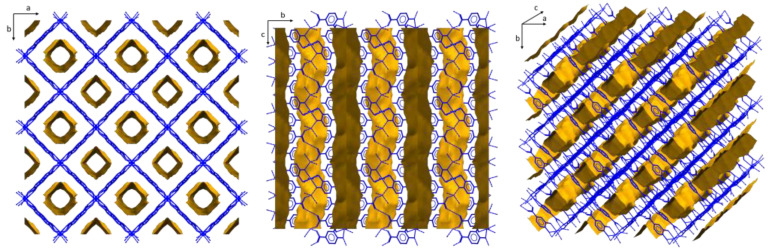
View of the packing of Co-MOF showing the solvent-accessible voids.

The growth of the 3D open framework leaves tubular microchannels of an approximate diameter of 9.1 Å (Figure S7), which are occupied by crystallization DMF and water molecules (Figure 2). The void volume corresponds to ca. 43% of the unit cell volume according to the geometrical calculation of the pore volume by PLATON-v1.18 program.

In our attempt to design multifunctional materials, we analysed Co-MOF magnetic properties and adsorptive capacity.

### 2.2. Magnetic Properties

**Figure 3:**
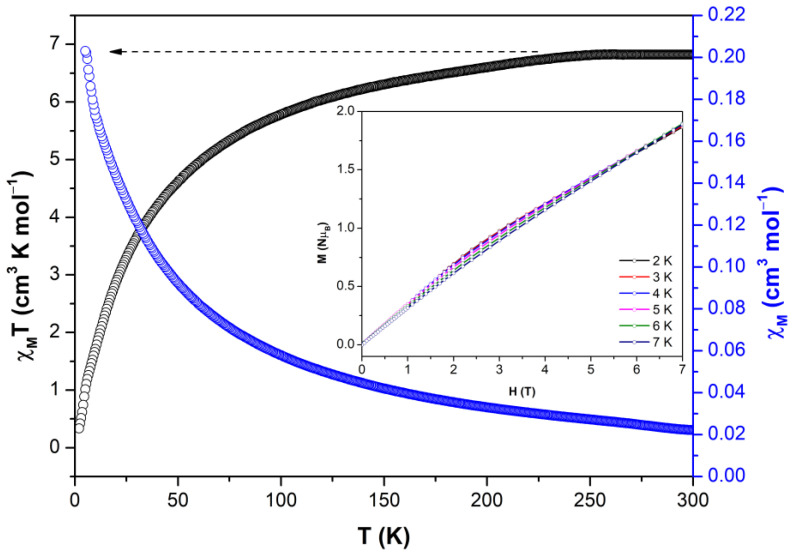
Temperature dependence of the χ_M_T product at 1000 Oe for Co-MOF Inset: M vs. H for Co-MOF 2–7 K. The lines are a guide to the eye.

Temperature-dependent magnetic susceptibility was measured on polycrystalline samples of Co-MOF in the range of 2–300 K and is shown in Figure 3. Upon cooling, the value of χ_M_T gradually decreases from 6.8 cm^3^·mol^−1^ K at 300 K to 4.7 cm^3^·mol^−1^ K at 50 K and then drops fast to 0.3 cm^3^·mol^−1^ K at 2 K. Below 8 K χ_M_T seems to suffer a slope change and to reach a maximum of at 5.75 K (1.15 cm^3^·mol^−1^ K), after which it subsequently drops to the minimum value at 2 K. This behaviour derives from the occurrence of antiferromagnetic interactions. In addition, at the highest temperature the magnetic value is higher than the expected spin-only value (1.875 cm^−3^·mol^−1^ K, S = 3/2), indicating a high *g* value (*g* > 2.0). The decrease of the χ_M_T at lower temperatures can be attributed to the combination of two factors: zero-field splitting of the ground state and/or antiferromagnetic exchange interactions [2,30]. The occurrence of a weak, but non-negligible maximum in the χ_M_T curve at 5.75 K seems to indicate that there is magnetic ordering in the compound, which may be attributed to a weak ferrimagnetic behaviour. Taking into account that crystal structure contains dimeric cores with a Co···Co distance of 3.304 Å, a short distance that may provide strong exchange interactions. In fact, previous compounds showing simultaneous µ-O and µ-carboxylate bridges between Co(II) ions are known to provide antiferromagnetic interactions [31]. However, as detailed by Xiao, Tong and coworkers in the characterization of the compound of [Co_2_(sdba)(Trp)_2_] formula [32], the antiferromagnetic coupling between an octahedrally distorted Co(II) ion (with an effective S = 1/2 at low temperature derived from the splitting of ^4^T_1g_ ground term into ^4^A_2_ and ^4^E levels) and a tetrahedral Co(II) ion (with a S = 3/2 because of the ground ^4^A_2_ term) may lead to a ferrimagnetic behavior and the occurrence of a net magnetic moment.

On the other hand, isothermal magnetization vs. applied field curves were measured at 2–7 K range showing a gradual increase with the applied external field without reaching a complete saturation of magnetization. This behaviour could be derived from the presence of significant anisotropy in the ground state and/or accessible low-lying excited states that are partially (thermally and field-induced) populated at this temperature range. In other words, the highest available field (7 T) may not be sufficient to fully depopulate the excited states to reach magnetization saturation for the studied complex [22,33]. The observed lack of saturation in these curves also supports the antiferromagnetic character of the compound [32].

**Figure 4:**
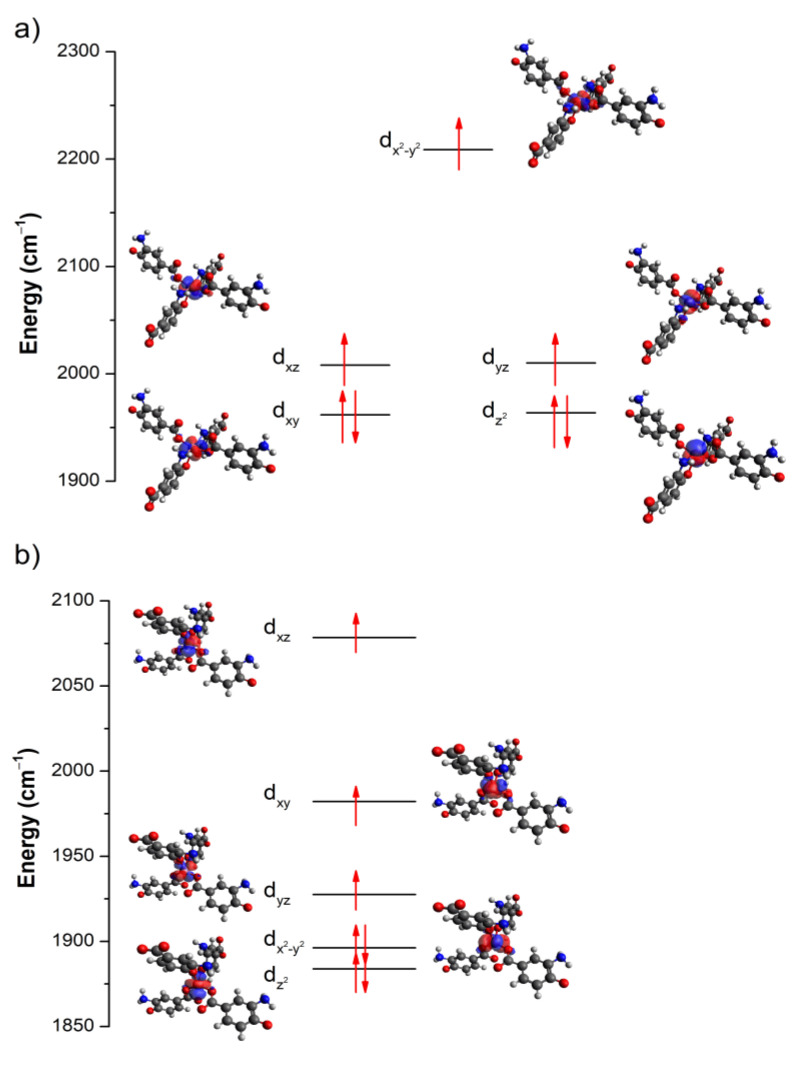
AILFT computed d-orbital splitting representation of the distorted (**a**) TPR and (**b**) Td coordination environments.

Additionally, CAS-SCF/NEVPT2 calculations were conducted over the two coexisting Co^II^ environments, distorted trigonal prism (TPR) and tetrahedral (Td), in an independent way (Figure 4). To that end, the models were taken from X-ray coordinates and slightly optimized in order to correct the effects derived from the structural disorder. Firstly, these calculations confirmed the high value of the gyromagnetic parameter (g = 2.38 and 2.27 for TPR and Td, respectively). According to the energetical distribution of the molecular orbitals, both Co(II) centres possess quite multideterminantal ground electronic configurations (Figure 4, and Table S6). On the one hand, the distorted TPR presents a dominant (dxy)^2^(dz^2^)^2^(dxz)^1^(dyz)^1^(dx^2^ − y^2^)^1^ configuration, with the dxy/dz^2^ and dxz/dyz pairs quasi-degenerated, which is not coincident with the expected orbital distribution for a real TPR environment, probably as a consequence of the high distortion of the coordination shell as confirmed by SHAPE. On the other hand, the second centre shows a (dz^2^)^2^(dx^2^ − y^2^)^2^(dyz)^1^(dxy)^1^(dxz)^1^ configuration, which reproduces more faithfully the energy order found in tetrahedral environments, except for the fact that orbital degeneracy is also broken in the present case. With regard to the magnetic anisotropy, the calculations give opposite signs for the values of the axial parameters as well as non-negligible rhombic contributions, which are consistent with other previously published works (*D* = −41.1 cm^−1^ and *E*/*D* = 0.20 for TPR, *D* = 24.0 cm^−1^ and *E*/*D* = 0.25 for Td environments) [34,35,36]. The major contributions to these parameters come from the ground-to-first and ground-to-second excited states, among which the origin of the rhombicity derives from the second and first excitations, respectively, for the distorted TPR and Td environments. Moreover, d_xy_ → d_xz_ and d_z_^2^ → d_xz_ and d_z_^2^ → d_yz_ and d_x_^2^_−y_^2^ → d_yz_ are the responsible transitions for the *zfs* occurring on the distorted TPR and Td centres. Taking into account that both centres coexist in the crystal, it is somewhat difficult to predict the final slow magnetic relaxation behaviour occurring in the compound.

**Figure 5:**
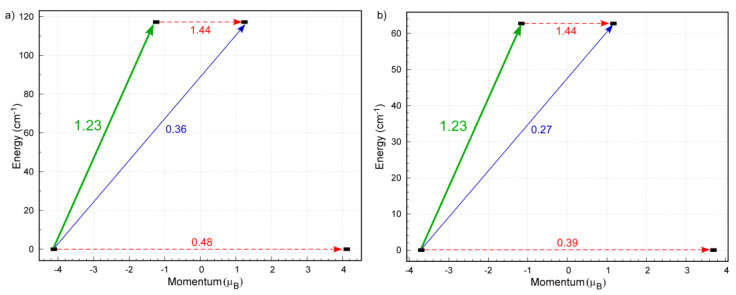
AILFT computed d-orbital splitting representation of the distorted (**a**) TPR; and (**b**) Td coordination environments.

To gain deeper insights into the potential relaxation pathways occurring in the compound, we computed the transition matrix elements and energies of the lowest-lying Kramers doublets of the Co(II) centre of both TPR and Td environments (see computational details for further explanation). First, it is worth highlighting that, owing to the disordered ligands around the Co(II) ion, both environments are built from the same ligands (they only differ by the coordinated donor atoms, which renders a six- or a four-connected environment) and hence, the calculated transition matrix elements are exactly the same in both environments (Figure 5). In fact, the only difference for both environments is the relative energy of the excited Kramers doublets, which lie at 117 and 63 cm^−1^ for the TPR and Td environments, respectively. As observed, the probability of QTM at the ground state is rather high (0.48 μB) owing to the significant rhombic contribution of the magnetic anisotropy. It is notable that the thermally assisted QTM becomes higher for the first excited state (1.44 μB). On the other hand, the probability for the Raman process seems to be higher than the pure Orbach relaxation (1.23 vs. 0.36 μB). All these facts may indicate that the compound may present a complex relaxation involving more than one mechanism.

With the aim of finding out if Co-MOF shows slow relaxation of the magnetization or not, dynamic alternating-current (*ac*) magnetic measurements were performed. Despite the expected large anisotropy of the Co^II^ ions, Co-MOF did not show any out-of-phase χ″_M_ signal under zero external field, which may be due to the fast resonant zero-field quantum tunnelling of the magnetization (QTM) through degenerate energy levels [21]. When the *ac* measurements were performed in the presence of an external *dc* field of 1000 Oe, Co-MOF showed weak frequency dependency, but with the maxima of χ_M_″ appearing below the instrument detection limit (Figures S8–S12). Thus, the energy barrier (*U_eff_*) and relaxation time (*τ*_0_) cannot be obtained via convectional Arrhenius method. However, if we assume that there is only one relaxation process, the Debye model (Equation (1)) could provide a rough estimation of *U_eff_* and *τ*_0_ values [37],

(1) lnχM″χM′=ln2πντ0+EakBT

yielding *U_eff_* value of 8.92 K and relaxation times (*τ*_0_) of 4.25·10^−8^ s^−1^ (see Figure S8). In this particular case, the contribution and exchange interaction of both tetrahedral and trigonal prismatic Co^II^ centres is taken into account to estimate the energy barrier of the magnetization reversal.

However, with the aim of isolating magnetic centres, Zn^II^ based magnetic dilution was carried out. Magnetic dilution involves the doping of Co^II^ paramagnetic centres into Zn^II^ diamagnetic matrix yielding heterometallic compounds. This strategy was shown to be an interesting approach to isolate paramagnetic centres, since it avoided magnetic exchange interactions, supressing long-range order, so that the material behaves as a single ion magnet (SIM) [38]. In particular, analysis of the more diluted compound [Co_0_._05_Zn_0.95_L]_n_, composed of 95% zinc in the metal stoichiometry) reveals slow magnetic relaxation according to the best fitted with Orbach and Raman relaxation processes Equation (2).

*τ*^−1^ = *τ*_0_^−1^exp(−*U*_eff_/*k*_B_*T*) + *BT*^n^ (2)

**Figure 6:**
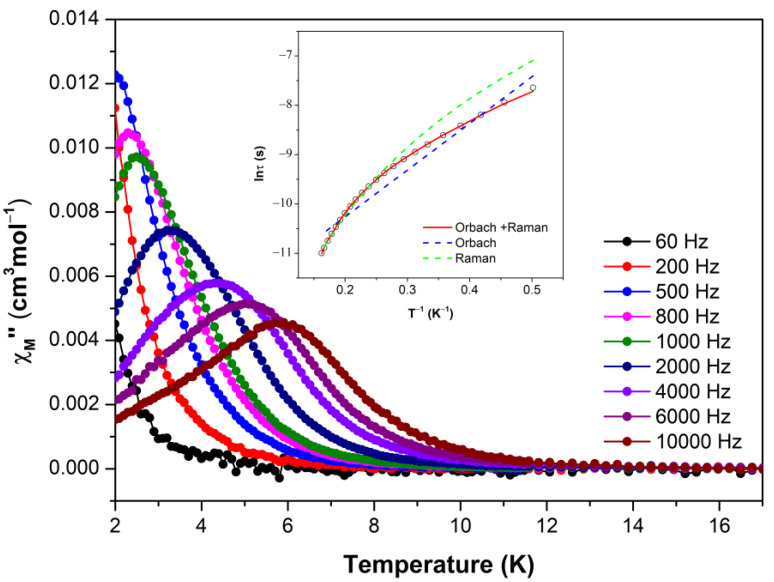
Temperature dependence of out-of-phase components of the ac susceptibility in a dc applied field of 1000 Oe for heterometallic [Co_0.05_Zn_0.95_L]_n_. Insets: Arrhenius plots. The blue line accounts for the best fit considering Orbach relaxation, the green line refers to Raman relaxation and the red line corresponds to the contribution of Orbach plus Raman relaxation.

Cole–Cole plots generated in the 2–6 K range display well-defined semicircles that may be fitted with the generalised Debye model [39], to estimate the nature of the relaxation processes. The obtained α values are within the range of 0.09(2 K)–0.04(6 K), suggesting a single mechanism involved in magnetic relaxation. However, when ln(τ)versus 1/T feature is plotted, the Arrhenius plots present a curved shape (Figure 6). Thus, fitting the high-temperature data to Orbach process gives τ_0_ = 8.44 10^−7^s^−1^ and *U_eff_* = 18.82 K. In any case, taking into account the shape of the curve, the relaxation times were fitted to an expression that considered the presence of simultaneous Orbach and Raman relaxation processes, giving the following set of data: τ_0_ = 2.09 10^−5^s^−1^, *U_eff_* = 6.31 K, *B* = 1.231 s^−1^·K^-n^ and *n* = 5.746. These facts are in agreement with the previous results obtained from the calculations, because the theoretical energy barrier (of 117 and 63 cm^−1^ for the TPR and Td environments) through the first excited state is clearly too high to imply that the Orbach mechanism is the unique relaxation pathway. Moreover, several examples in bibliography have shown that either tetrahedral [38,40,41] and trigonal prismatic [21,22,42,43] Co^II^ environments tend to relax by multiple relaxation pathways where the relaxation data should be modelled with accounting for the contributions from direct, QTM, Raman and Orbach relaxation processes. In our case, as the Co^II^ environment is supposed to be ideally isolated wherein the network and the contribution of both relaxation modes corresponding to its centre have been considered for the best fitting.

Interestingly, the presence of Zn^II^ in the heterometallic samples imbues them with photoluminescent properties. Motivated by this, we decided to explore photoluminescent properties of [Co_x_Zn_1-x_L]_n_ heterometallic compounds as well the pure Zn^II^ based material. To that end, we took advantage of the fact that these compounds were isostructural to the a zinc-based counterpart previously reported in bibliography [11]. That Zn^II^ based metal-organic framework described by Zhang et al. had proved to have an extraordinary acid–based resistance and was able to efficiently separate acetylene from C_2_H_2_/CO_2_ mixtures under ambient conditions.

For the synthesis of heterometallic compounds, several proportions of Zn^II^ to Co^II^ combinations were employed. (See Table S1 for more details). Chemical and physical characterization as well as powder XRD data (Figures S3 and S4) confirmed the success of partial replacement in the resulting heterometallic counterparts. Additionally, we further confirmed the presence of both metals in single crystals by EDX mapping, in which the final proportions in the counterpart show slight deviations from those expected but within the experimental error known for this semi-quantitative technique (see Table S1 and Figure S1 in the ESI).

### 2.3. Photoluminescent Properties

**Figure 7:**
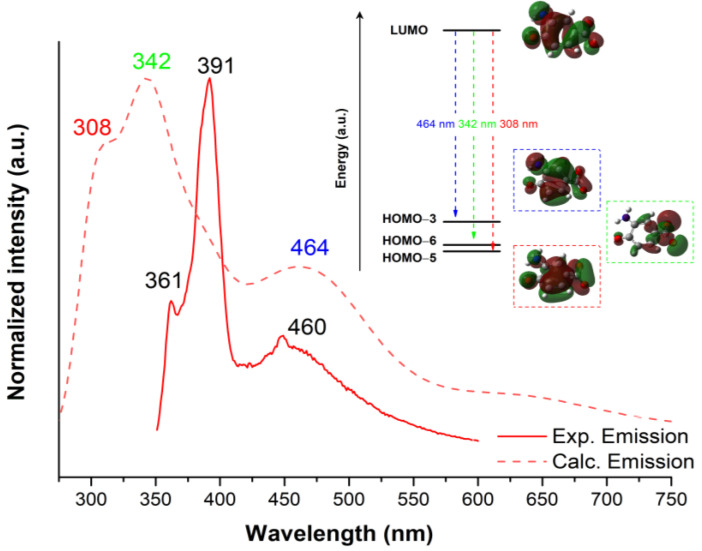
Room temperature time-dependent density-functional theory (TD-DFT) computed (dashed lines) and experimental (solid lines) photoluminescence under λ_ex_ = 330 nm polycrystalline homometallic Zn^II^ complex. The insets show the most representative molecular orbitals involved in the electronic transitions.

The solid-state photoluminescence spectra were recorded at ambient temperature for polycrystalline homometallic (Zn^II^ and Co^II^) and [Co_x_Zn_1-x_L]_n_ heterometallic samples. We first decided to explore the homometallic Zn^II^ emission capacity, given that group 12 metals are known to be particularly suitable for their use in photoluminescence, contrarily to what occurs for Co^II^ [44]. The closed-shell electronic configuration affords no d–d transitions, which could enhance ligand-centred (LC) emissions [45]. Furthermore, the presence of these ions may also promote ligand-to-metal charge transfer (LMCT), as metal ions possess empty orbitals that can be populated in the excited state, and therefore the PL emission may be modulated with regard to the ligand-centred (LC) emissions [46]. Upon excitation with 330 nm light, the zinc-based compound shows three maxima peaking at 361, 391 and 460 nm, among which the second one dominates the emission spectrum (Figure 7). The excitation spectrum focusing on the main emission line exhibits several absorption bands located in the ultraviolet region with four maxima at around 288, 307, 322 and 332 nm, which resemble the excitation spectra found for the previously reported ligand [47]. Therefore, the observed bands can be attributed to inner π–π* transitions occurring in the aromatic ring of the 3-amino-4-hydroxybenzoic acid ligand. In order to gain deeper insight into the emission mechanism, TD-DFT calculations were performed on a suitable model of a homometallic Zn^II^ compound. The calculated spectra reprocess the experimental one fairly well, indicating that the process is conducted by three main transitions between the molecular orbitals depicted in Error! Reference source not found. Nonetheless, a shift of around 50 nm is observable in the first two transitions, thus correlating the transition calculated at 308 nm to the experimental 361 nm transition and the calculated 342 nm transition to the 391 nm experimental transition, respectively. The electron density of HOMO orbitals HOMO-5 and HOMO-3 is extended over the aromatic ring, suggesting a π orbital, whereas the LUMO orbital features a π* character. Thus, it can be confirmed that the transitions involved in the photoluminescence are mainly of a π*←π nature induced by a ligand-centred emission, as further confirmed by the agreement of the experimental data and TD-DFT calculations.

The emission and excitation spectra of Co-MOF (based on the cobalt counterpart) show similar patterns (see Figure S14) with much less emission intensity due to the quenching exerted by this ion.

**Figure 8:**
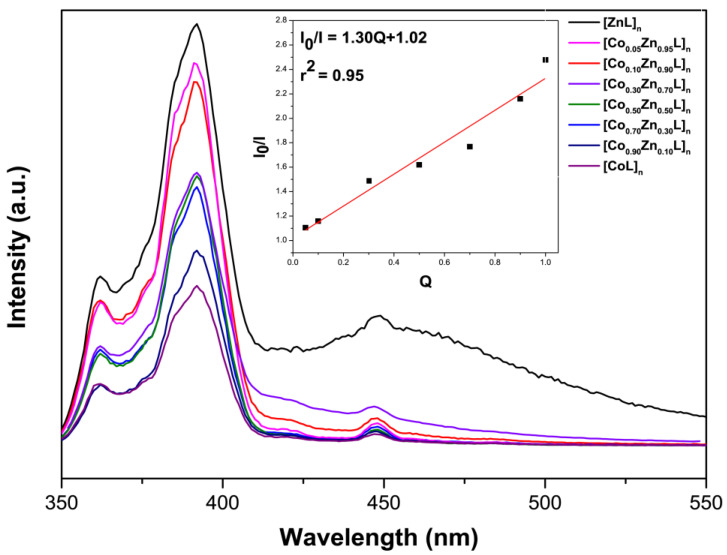
Fluorescence spectra of homometallic Zn^II^ and Co^II^ compounds and [Co_x_Zn_1-x_L]_n_ (where x= 0.9–0.1 stands for the proportion of Zn in the formula) at RT (λ_ex_ = 330 nm). Inset graph: Stern–Volmer plot with regard to the relative intensity of the main emission band.

With the aim of integrating the photoluminescence properties into the magnetic Co-MOF, the heterometallic mixtures were further studied. The main emission bands of [Co_x_Zn_1-x_L]_n_ materials keep the same shape but are somewhat structured compared to homometallic compounds. Additionally, the minor shoulder peaking at λ_em_ = 448 nm presents a relatively lower intensity for the heterometallic materials than for the zinc analogue. No remarkable shift of the main bands is observed between homo- and heterometallic compounds. In fact, the inset in Figure 8 reveals that there is a linear relationship between the intensity of the main signal (peaking at 392 nm) and the Co^II^ proportion, since it acts as a luminescent quencher. Thus, the quenching efficiency was estimated applying the Stern–Volmer Equation (3) which correlates the quencher concentration with the fluorescence decrease by a quencher constant.

(3) $$I/I_{0}=1+{\;k}_{\mathrm{sv}}Q$$

being I the fluorescence intensity of heterometallic compound, I_0_ the reference fluorescence intensity of the Zn counterpart and Q the quencher (Co^II^) concentration.

The linear fitting of Equation (3) gives a value of *k_sv_* value of 1.30(0.1), meaning that the quenching capacity of cobalt(II) ions is relatively low and that the resulting materials may be considered to be bifunctional. Among all the studied samples, that containing the lowest proportion of Co(II), namely [Co_0.05_Zn_0.95_L]_n_, is probably the most interesting one, since it presents both single-ion magnet and photoluminescence properties.

## 3. Materials and Methods

### 3.1. Preparation of Complexes

All chemicals were of reagent grade and used as commercially obtained. 3-amino-4-hydroxybenzoic acid ligand (H_3_L, C_7_H_7_NO_3_, 97% of purity) was purchased from Fluorochem and cobalt(II) nitrate hexahydrate (99% of purity, Merck, Boston, MA, USA) were employed as metallic precursor.

Synthesis of {[CoL]·0.5DMF·H_2_O}_n_

- Synthetic pathway to obtain single crystals

In a 6 mL screw-capped vial, 0.010 g (0.0625 mmol) of 3-amino-4-hydroxybenzoic acid organic linker and an equivalent of Co(NO_3_)_2_·6H_2_O, 0.018 g (0.0625 mmol), were weighted. Then, 4 mL DMF/0.3 mL EtOH/0.3 mL MeCN/0.1 mL H_2_O solvent-mixture was added to the vial, which was sealed and introduced to the oven at 140 °C for 2 h to give rise rod-shaped single crystals. Single-crystal X-ray structure determination, elemental analysis (EA) and TGA (Figure S5 and S6) confirm the title formula, C_17_H_21_Co_2_N_3_O_9_. EA: calcd: C, 38.58; H, 4.00; N, 7.94; found: C, 38.77; H, 3.98; N, 8.00. TGA data for loss of half of a DMF molecule and H_2_O: calcd: 20.7%, found: 19.0%. In addition to the elemental analyses, the purity of the sample was checked by powder X-ray diffraction (Figure S3, ESI).

- Synthetic pathway to obtain polycrystalline powder

3-amino-4-hydroxybenzoic acid ligand (0.2 g, 1.20 mmol) was dissolved in 2 mL of DMF/2 mL of H_2_O solvent mixture. Co(NO_3_)_2_·6H_2_O (0.35 g, 1.2 mmol) was dissolved in 1 mL of DMF/ 1 mL of H_2_O solvent mixture. The metal solution was added dropwise into the ligand solution under magnetic stirring until a purple precipitate was formed. The product was collected by filtration and washed with DMF and H_2_O. 0.112 g (yield 44%). Sample purity was checked by powder X-ray diffraction.

### 3.2. Physical Measurements

Elemental analyses (C, H, N) were performed on a Leco CHNS-932 microanalyser. Infrared (IR) spectra (400–4000 cm^−1^) were recorded on a Nicolet FT-IR 6700 spectrometer in KBr pellets (Figure S2). Diffuse reflectance measurements were made on polycrystalline samples of Zn, Co metallic and heterometallic [Co_x_Zn_1-x_L]_n_ compound in a UV/Vis Shimadzu spectrophotometer (Figure S13). These spectra were recorded at room temperature with BaSO_4_ as reference material. Thermogravimetric analyses (TG/DTA) were performed on a TG-Q500 TA Instruments thermal analyser from room temperature to 800 °C under a synthetic air atmosphere (79% N_2_/21% O_2_) at a heating rate of 10 °C min^−1^. Magnetic susceptibility measurements were performed on polycrystalline samples of the complexes with a Quantum Design SQUID MPMS-7T susceptometer at an applied magnetic field of 1000 G. The susceptibility data were corrected for diamagnetism estimated from Pascal’s tables [48], the temperature-independent paramagnetism and magnetisation of the sample holder. The *ac* measurements were performed on a physical property measurement system quantum design model 6000 magnetometer under a 3.5 G *ac* field and frequencies ranging from 60 to 10,000 Hz. Photoluminescence (PL) measurements were carried out on crystalline samples at room temperature using a Varian Cary-Eclipse fluorescence spectrofluorimeter equipped with a Xe discharge lamp (peak power equivalent to 75 kW), Czerny–Turner monochromators, and an R-928 photomultiplier tube. For the fluorescence measurements, the photomultiplier detector voltage was fixed at 600 V, and the excitation and emission slits were set at 5 and 5 nm, respectively.

### 3.3. Single-Crystal X-ray Diffraction (SCXRD)

SCXRD data of suitable single crystals of Co-MOF was collected at 100(2) K on a Bruker VENTURE area detector equipped with graphite monochromated Cu_Kα_ radiation (*λ* = 1.54178 Å). Data reduction was performed with the APEX3 program [49] whereas absorption correction was done with SADABS [50]. The structures were solved by direct methods and refined by full-matrix least-squares with SHELXL-2018 [51] and refined by full-matrix least-squares on F^2^ including all reflections, by employing the WINGX crystallographic package [52]. All hydrogen atoms were located in difference Fourier maps and included as fixed contributions riding on attached atoms with isotropic thermal displacement parameters 1.2 times those of their parent atoms for the 3-amino-4-hydroxybenzoate ligand. The ligand is disordered about a twofold rotation axis over two sites with equal occupancy and is essentially superimposed upon itself. Details of the structure determination and refinement Co-MOF are summarised in Table S2. Deposition Number 2235336 contain the supplementary crystallographic data for this paper. These data are provided free of charge by the joint Cambridge Crystallographic Data Centre and Fachinformationszentrum Karlsruhe Access Structures service www.ccdc.cam.ac.uk/structures (accessed on 4 February 2023).

The powder XRD patterns were collected on a Phillips X’PERT powder diffractometer with Cu_Kα_ radiation (*λ* =1.5418 Å) over the range 5 < 2θ < 50ᵒ with a step size of 0.026ᵒ and an acquisition time of 2.5 s per step at 25 °C. Indexation of the diffraction profiles was made by means of the FULLPROF program (pattern-matching analysis) [53] on the basis of the space group and the cell parameters found for isostructural compounds by means of single-crystal XRD. The unit-cell parameters obtained in the final refinement are listed in the Supporting Information.

Variable-temperature PXRD were recorded on a PANalytical X’Pert Powder diffractometer (Cu-Kα1,2 X-radiation, λ_1_ = 1.540598 Å; λ_2_ = 1.544426 Å) under air, equipped with a PIXcel 1D detector, a flat-plate sample holder in a Bragg–Brentano para-focusing optics configuration (40 kV, 50 mA), and a high-temperature Anton Paar HKL 16 chamber controlled by an Anton Paar 100 TCU unit. Intensity data were collected in the continuous mode (ca. 100 s data acquisition) in the angular range ca. 5 ≤ 2θ° ≤ 35.

### 3.4. Computational Details

Gaussian 16 package [54] was employed for optimizing the TPR and Td coordination excerpts, starting from X-ray coordinates and performing optimizations at DFT level of theory with UB3LYP functional [55] and the TZV basis set for the cobalt atom [56] and the 6-31G** basis set for the rest of non-metal atoms [57]. Magnetic coupling in compounds 4Cu and 5Cu was analysed using broken-symmetry formalism [58,59] and also by means of CASSCF calculations (CAS (2,2) setup), using ORCA software suite in both cases (version 5.0.2) [60,61]. These single point calculations were conducted in ab initio calculations for both models and were implemented in ORCA to estimate *zfs* parameters for the optimized models. Calculations with state-average complete active space self-consistent field (SA-CASSCF) method were performed incorporating the five d-orbitals and seven electrons. B3LYP functional [62,63] using def2-TZVP basis sets for all atoms and def2-QZVPP for the metal atoms, recontracted for zeroth-order regular approximation (ZORA) relativistic approximation were employed [64,65,66,67]. RIJCOSX approximation with appropriate auxiliary basis sets (def2/J) [66] were employed for all calculations. Ten quartets and forty doublets were included in the calculations [68]. NEVPT2 calculations were performed on SA-CASSCF converged wave functions to take into account the dynamic correlation [69], a strategy successfully used earlier to obtain accurate estimations of the *zfs* parameters [68,70]. Spin Hamiltonian parameters were also calculated on top of the converged CASSCF energies results by means of SINGLE_ANISO code as implemented in ORCA, including spin–orbit coupling (SOC) effects and a subsequent quasi-degenerate perturbation theory (QDPT) step [71,72].

## 4. Conclusions

In conclusion, we have successfully synthesised a novel Co-MOF) by solvothermal reaction. [CoL]_n_ possesses a 3D porous network with the **pts** topology, which is constructed by the connection of Co(NO_3_)_2_·6H_2_O and 3-amino-4-hydroxybenzoic acid ligand. The magnetic properties of Co-MOF were studied by CASSCF/NEVPT2 calculations, in spite of the coexistence of two disordered Co(II) centres with hexa- and tetra-coordinated environments. These model excerpts present similar *zfs* parameters in magnitude but with opposed signs, a fact that may be the reason for the low-energy barrier estimated for the field-induced slow magnetic relaxation. In the attempt to add multifunctionality to Co-MOF), a diamagnetic-matrix based dilution allowed us to obtain a material with a single ion magnet behaviour, showing a magnetic barrier for the reversal of magnetization of *U_eff_* = 6.31 K. Additionally, the photoluminescent studies of heterometallic Zn^II^-Co^II^ and homometallic isostructural Zn^II^ materials showed a linear response in the fluorescent intensity decrease with respect to the Co^II^ quencher incorporation in the structure. Overall, this work will prompt further designs of a multifunctional porous Co^II^-based MOF that is able to show single-ion magnet behaviour in a diluted matrix as well as emissive properties.

## Data Availability

The data presented in this study are available in this article or Supplementary Material.

## Supplementary Materials

The following are available online at https://www.mdpi.com/article/10.3390/molecules28041846/s1, Figure S1. EDS mapping of a representative portion of the [Co_0.05_Zn_0.95_L]_n_ heterometallic material. Figure S2. Figure of the infrared spectra of the ligand and Co-MOF. Figure S3. Figure of the pattern matching analysis and experimental PXRD for Co-MOF. Figure S4. Experimental PXRD of [Co_x_Zn_1-x_L]_n_ heterometal samples. Figure S5. Figure of TG/DTA analysis of Co-MOF (left; up, as synthesised, down, after solvent exchange with MeOH) and figure of the experimental PXRD for Co-MOF before and after solvent exchange with MeOH (right). Figure S6. Thermal evolution of Co-MOF. Figure S7. View along a (left), b (middle) and c (right) axis of Co-MOF (down). Figure S8. Temperature dependence of in-phase (blue) and out of phase (red) components of the ac susceptibility in a dc applied field of 1000 Oe for Co-MOF. Figure S9. Plot of ln(χ_M_”/χ_M_’) versus 1/T at 10,000 Hz for Co-MOF under an applied field of 1000 Oe. The solid lines represent the linear fit with ln(χ_M_”/χ_M_’) = ln(2πντ_0_) + E_a_/k_B_T. Figure S10. Temperature dependence of in-phase components of the ac susceptibility in a dc applied field of 1000 Oe for [Co_0.05_Zn_0.95_L]_n_. Figure S11. Cole-Cole plots in a dc applied field of 1000 Oe for [Co_0.05_Zn_0.95_L]_n_. Figure S12. Variable-temperature frequency dependence of the χ_M_” signal under 1000 Oe applied field for [Co_0.05_Zn_0.95_L]_n_. Solid lines represent the best fitting of the experimental data to the Debye model. Figure S13. Diffuse reflectance of 3-amino-4-hydroxybenzoic acid ligand, homometallic Co-MOF, and Zn compounds and heterometallic [Co_0.05_Zn_0.95_L]_n_ heterometallic sample. Figure S14. Figure caption of the experimental room temperature photoluminescence excitation and emission spectra under λ_em_ = 391 nm and λ_ex_ = 330 nm, respectively for Co-MOF and isostructural Zn^II^ homometallic counterpart. Table S1. Doping percentage, mmols and corresponding weight used in the synthesis of [Co_x_Zn_1-x_L]_n_ heterometal samples. Table S2. Crystallographic data and structure refinement details of Co-MOF. Table S3. Table of the selected bond lengths (Å) and angles (°) for Co-MOF. Table S4. Table of the continuous Shape Measurements for the CoO_4_ coordination environment. Table S5. Table of the continuous Shape Measurements for the CoN_2_O_4_ coordination environment. Table S6. Calculated orbital geometry related parameters.
