# Supplementary material for: Slow Magnetic Relaxation and Modulated Photoluminescent Emission of Coordination Polymer Based on 3-Amino-4-hydroxybenzoate Zn and Co Metal Ions
Source: Molecules. 2023 Feb 15;28(4):1846. doi: 10.3390/molecules28041846 (PMC9964557; doi:10.3390/molecules28041846)
Supplement: Supplementary file 1 [file molecules-28-01846-s001.zip › molecules-2185470-supplementary.pdf]

Supporting Information for:

Slow magnetic relaxation and modulated photoluminescent emission of coordination polymer based on 3-amino-4-hydroxybenzoate Zn and Co metal ions

Estitxu Echenique-Errandonea,[1] Sara Rojas,[2] Javier Cepeda,[1] Duane Choquesillo-Lazarte,[3] Antonio Rodríguez-Diéguez*,[2] and José M. Seco*,[1]

[1] Departamento de Química Aplicada, Facultad de Química, Universidad del País Vasco UPV/EHU, Paseo Manuel Lardizabal, nº 3, 20018, Donostia-San Sebastián, Spain. Email: josemanuel.seco@ehu.eus

[2] Departamento de Química Inorgánica, Facultad de Ciencias, Universidad de Granada, Av. Fuentenueva S/N, 18071 Granada, Spain. Email: antonio5@ugr.es

[3]. Laboratorio de Estudios Cristalográficos, IACT (CSIC-UGR), Avda. de las Palmeras 4, Armilla, Granada, Spain

Table of Contents:

1. Chemical characterization of compounds 3
2. FT-IR spectroscopy 5
3. Crystallographic data 6
4. Selected bond lengths and angles data 7
5. Powder X-ray diffraction analysis 9
6. Continuous Shape Measurements 10
7. Thermal analysis 11
8. Thermal evolution 12
9. Additional views of the structure 13
10. *Ac* magnetic susceptibility measurements 14
11. Diffuse reflectance measurements 18
12. Photoluminescence properties 19

1. Chemical characterization of compounds

Synthesis of $[Co_xZn_{1-x}L]_n$ where x is doping percentage

The heterometallic compounds were synthesis by following previously described general procedure, but with an appropriate molar amounts of $Co(NO_3)_2{\cdot}6H_2O$ and $Zn(NO_3)_2{\cdot}6H_2O$ that accounted for a total amount of 1.2 mmol. to prepare the solution of metal salt. For more details, see Table S1 The resulting purple polycrystalline samples (with variable colour intensity dependant of the Co^{II} concentration) were filtered off and washed several times with water and dimethylformamide. The purity of each doped sample was checked by PXRD. The purity of each doped sample was checked by PXRD. .3-amino-4-hydroxybenzoic acid ligand (H_3L, $C_7H_7NO_3$, 97 % of purity) was purchased from Fluorochem. Zinc nitrate hexahydrate (99 %, Alfa Aesar) and cobalt(II) nitrate hexahydrate (99 % of purity, Merck) were employed as metallic precursors.

Table S1. Doping percentage, mmols and corresponding weight used in the synthesis of $[Co_xZn_{1-x}L]_n$ heterometallic samples.

**% Co**	mmol (g)	**%Zn**	mmol (g)
**0**	-	**100**	1.20 (0.400)
**5**	0.06 (0.018)	**95**	1.14 (0.390)
**10**	0.12 (0.035)	**90**	1.08 (0.320)
**30**	0.36 (0.105)	**70**	0.84 (0.25)
**50**	0.60 (0.175)	**50**	0.60 (0.178)
**70**	0.84 (0.244)	**30**	0.36 (0.107)
**90**	1.08 (0.314)	**10**	0.12 (0.0357)
**100**	1.20 (0.350)	**0**	-

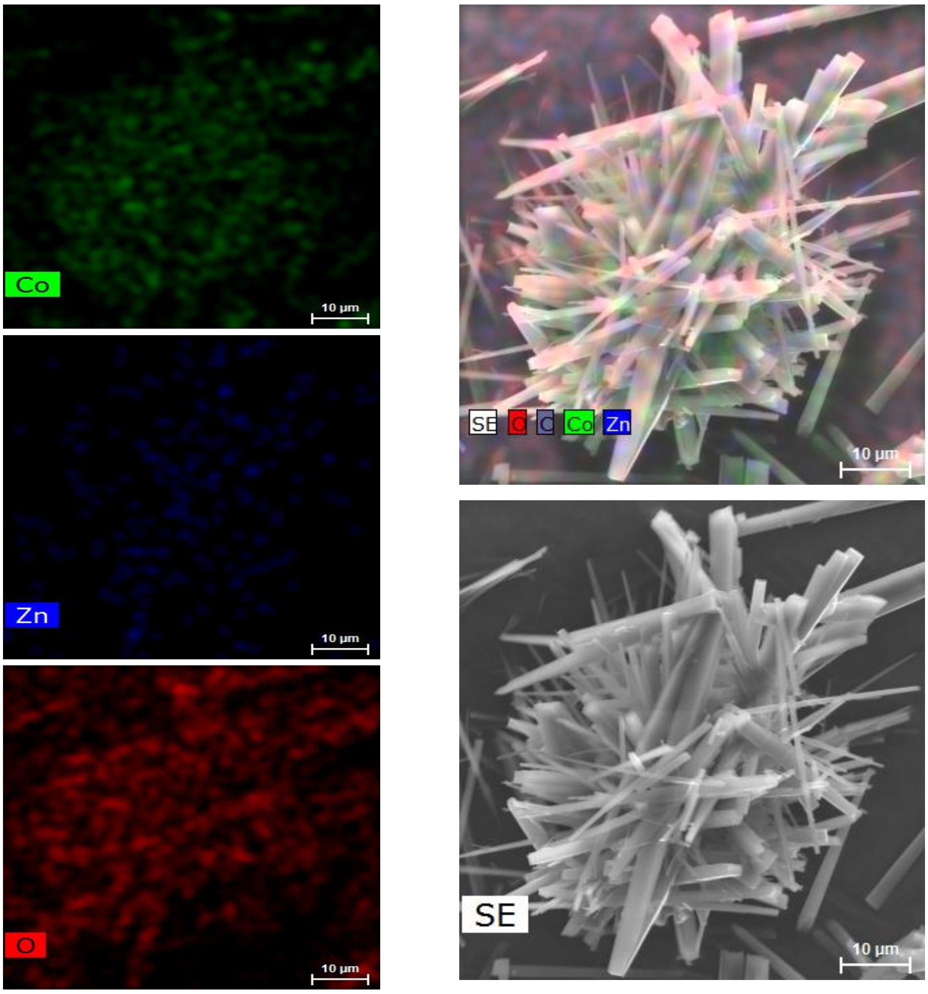

Figure S1. EDS mapping of a representative portion of the $[Co_{0.05}Zn_{0.95}L]_n$ heterometallic material.

EDS (energy dispersive X-ray spectroscopy) data and SEM mapping images were recorded on the Hitachi SU-70, working at 15 kV and using either a Bruker Quantax 400 or an Esprit 1.9 EDS microanalysis system.

Element	Series	unn. C [wt.%]	norm. C [wt.%]	Atom. C [at.%]	Error (3 Sigma) [wt.%]
Oxygen	K-series	28.14	28.19	28.42	13.48
Carbon	K-series	48.59	48.67	65.36	20.81
Zinc	K-series	4.14	4.14	1.02	1.07
Cobalt	K-series	18.96	18.99	5.20	2.25
	Total:	99.83	100.00	100.00	

Despite the fact that the resulting proportion obtained for these single crystals differ slightly to those expected, it must be taken into account that this is a semi-quantitative analysis and that errors are within the typical range.

2. FT-IR spectroscopy

FTIR spectra of Co-MOF exhibit broad and intense band around 3439 cm^{-1} that corresponds to the O–H bond vibration of the of 3-amino-4-hydroxybenzoate free ligand, The bands between 3331 cm^{-1} and 2928–2857 cm^{-1} can be attributed to aromatic ring's C–H bond vibrations of the ligand. The intense vibrations in the 1668–1430 cm^{-1} region are referred to both the asymmetric stretching vibrations of the carboxylate groups and the aromatic C–C and C–N bonds. The symmetric stretching vibrations of the carboxylate groups appear in the lower range of 1391–1297 cm^{-1}. The remaining bands that are found at lower frequency can be attributed to the distortions originated in the aromatic ring and the carboxylate groups of the ligands. The vibration bands of the M–O and M–N bonds are observed below 670 cm^{-1}.

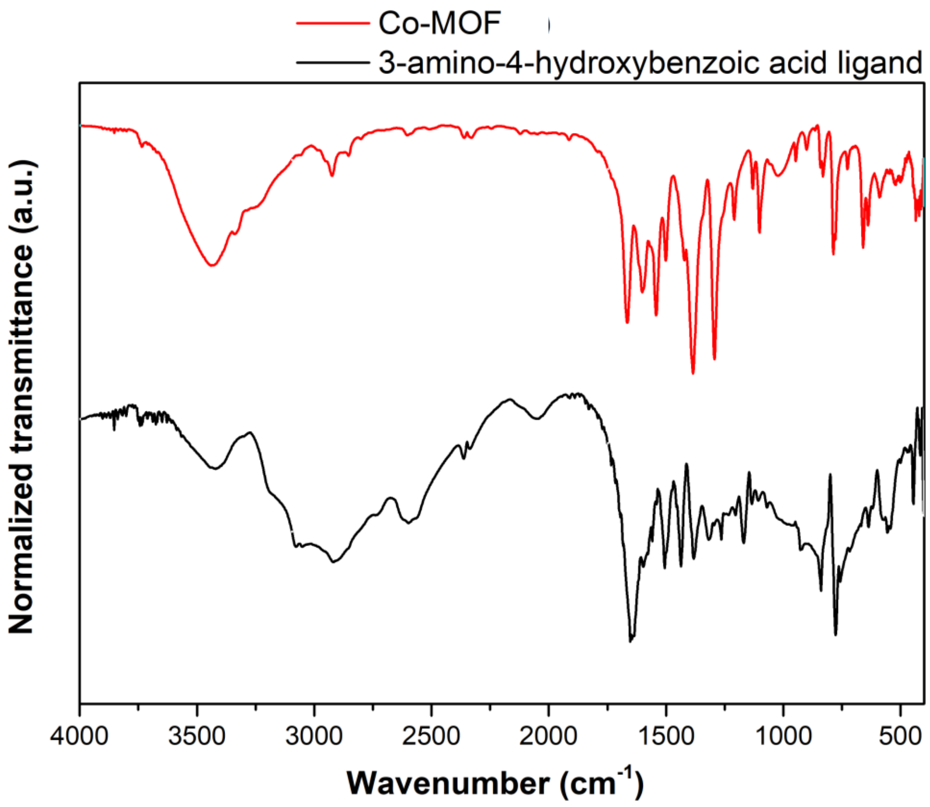

Figure S2. Figure of the infrared spectra of the ligand and Co-MOF**.**

3. Crystallographic data

Table S2. Crystallographic data and structure refinement details of Co-MOF**.**

Co-MOF	
Formula	$C_7H_5NO_3Co$
M_r	210.05
Crystal system	tetragonal
Space group (no.)	*$P4_2$(77)*
a(Å)	9.1366(2)
b(Å)	9.1366(2)
c(Å)	6.6080(3)
α(°)	90
β(°)	90
γ(°)	90
V(Å^3)	551.62(3)
Z	2
ρ_{calc}g/cm^3	1.265
μ/mm^{-1}	12.007
F(000)	210.0
Crystal size/mm^3	0.700 × 0.124 × 0.080
Radiation	CuKα (λ = 1.54178)
2θ range for data collection/°	16.556 to 132.752
Index ranges	-10 ≤ h ≤ 10, -10 ≤ k ≤ 10, -7 ≤ l ≤ 7
Reflections collected	6346
Independent reflections	933 [R_{int} = 0.0284, R_{sigma} = 0.0180]
Data/restraints/parameters	933/70/106
Goodness-of-fit on F^2	1.152
Final R indexes [I>=2σ (I)]	R_1 = 0.0562, wR_2 = 0.1643
Final R indexes [all data]	R_1 = 0.0716, wR_2 = 0.1833
Largest diff. peak/hole / e Å^{-3}	0.76/-0.29

4. Selected bond lengths and angles data

Table S3. Table of the selected bond lengths (Å) and angles (°) for Co-MOF**.**

Co-MOF		
Co1	O2	1.8610(157)
	O3	1.9145(173)
	O1	1.9367(242)
	O1	2.0802(241)
	N1	2.4091(169)
	O2	3.1768(204)
	Co1	3.304(14)
	O3	3.3833(160)
	N1	3.5469(246)

Atom 1	Atom 2	Atom 3	Angle 2,1,3 [º]
Co1	O2	O2	139.187(637)
	O2	O3	94.161(649)
	O2	O3	95.754(649)
	O2	O1	106.728(634)
	O2	O1	105.999(634)
	O2	O1	36.326(586)
	O2	O1	102.862(572)
	O2	N1	35.482(597)
	O2	N1	174.648(608)
	O2	O2	106.943(625)
	O2	O3	36.354(555)
	O2	O3	102.835(569)
	O2	N1	75.025(610)
	O2	N1	74.452(610)
	O2	O2	106.943(624)
	O3	O3	151.303(735)
	O3	O1	111.754(615)
	O3	O1	39.549(648)
	O3	O1	101.828(571)
	O3	O1	102.089(570)
	O3	N1	85.908(764)
	O3	N1	86.702(766)
	O3	O2	108.950(533)
	O3	O2	42.354(546)
	O3	O3	101.962(494)
	O3	N1	145.330(617)
	O3	N1	63.367(650)
	O1	O1	72.205(950)
	O1	O1	132.495(760)

O1	O1	132.495(761)
O1	N1	77.979(585)
O1	N1	77.765(584)
O1	O2	2.823(657)
O1	O2	69.404(785)
O1	O3	132.406(585)
O1	O3	132.616(586)
O1	N1	102.914(694)
O1	N1	175.118(651)
O1	O1	66.541(828)
O1	N1	71.802(491)
O1	N1	138.342(510)
O1	O2	134.064(643)
O1	O2	134.598(640)
O1	O3	0.134(467)
O1	O3	66.515(628)
O1	N1	50.778(611)
O1	N1	50.949(612)
N1	N1	149.856(553)
N1	O2	77.203(570)
N1	O2	77.692(570)
N1	O3	71.828(489)
N1	O3	138.316(482)
N1	N1	101.321(585)
O2	O2	66.605(494)
O2	O3	133.980(407)
O2	O3	134.715(407)
O2	N1	105.715(470)
O2	N1	172.315(478)
O3	O3	66.490(319)
O3	N1	50.654(394)
O3	N1	51.046(394)
N1	N1	81.966(489)

5. Powder X-ray diffraction analysis

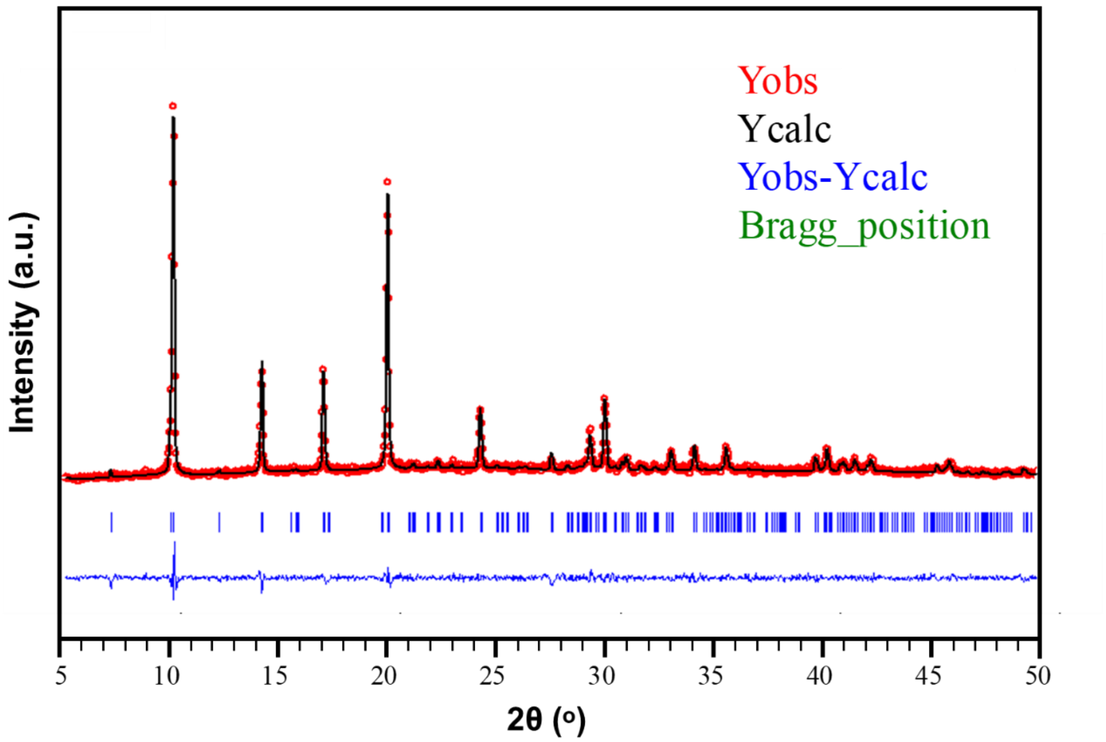

Figure S3. Figure of the pattern matching analysis and experimental PXRD for Co-MOF.

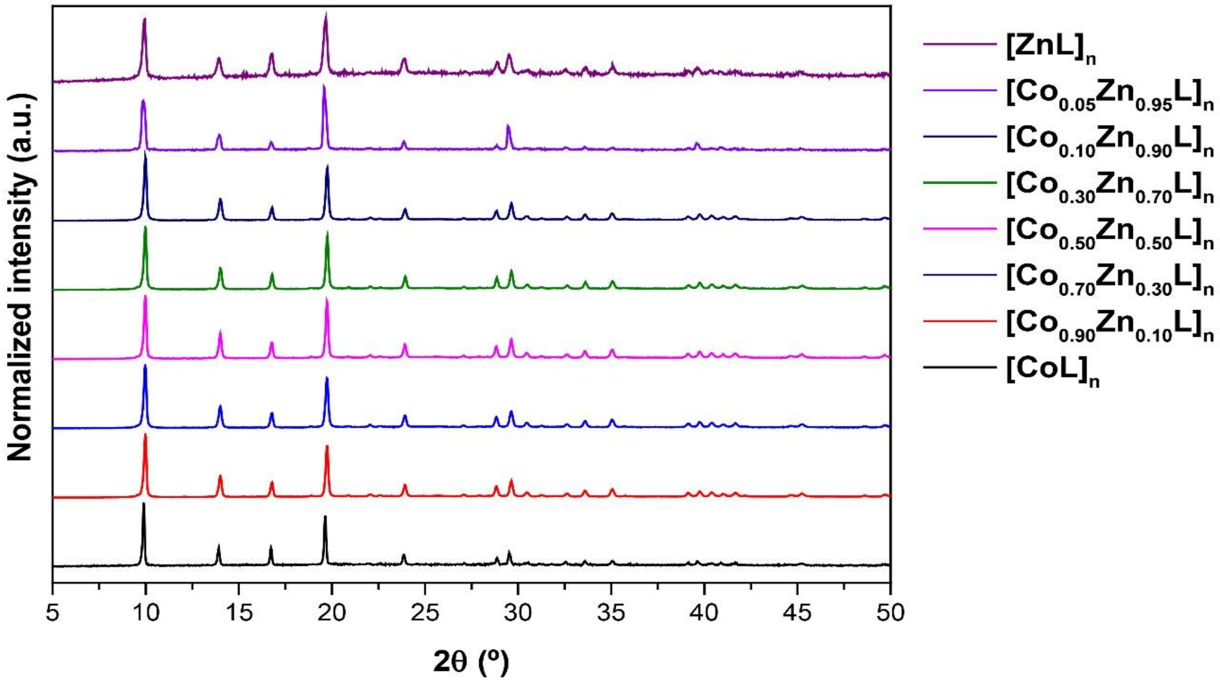

Figure S4. Experimental PXRD of $[Co_xZn_{1-x}L]_n$ heterometallic samples.

6. Continuous Shape Measurements

CShMs for the coordination environment of compounds **1**. The lowest SHAPE values for each ion is shown highlighted in grey, indicating best fits.

Table S4. Table of the continuous Shape Measurements for the CoO_4 coordination environment

SP-4	D_{4h}	Square
T-4	Td	Tetrahedron
SS-4	C_{2v}	Seesaw
vTBPY-4	C_{3v}	Vacant trigonal bipyramid

Complex	SP-4	T-4	SS-4	vTBPY-
	30.170	**1.878**	4.528	2.270

Table S5. Table of the continuous Shape Measurements for the CoN_2O_4 coordination environment

HP-6	D_{6h}	Hexagon
PPY-6	C_{5v}	Pentagonal pyramid
OC-6	O_h	Octahedron
TPR-6	D_{3h}	Trigonal prism
JPPY-6	C_{5v}	Johnson pentagonal pyramid J2

Complex	HP-6	PPY-6	OC-6	TPR-6	JPPY-6
	34.045	12.509	12.883	**7.490**	15.585

7. Thermal analysis

Thermogravimetric analyses have been performed over polycrystalline sample in Co-MOF in order to check the stability of the product. The TG curves has been collected for Co-MOF before and after solvent exchange with MeOH. This procedure has been carried out suspending three times the material in MeOH for an hour. Solvent exchange procedure has been accomplished as an approach to replace lattice-solvent molecules (dimethylformamide and water) to ease material activation to posteriorly analyse its adsorptive-capacity. Powder X-ray diffraction confirmed that Co-MOF_MeOH remains stable after solvent exchange with MeOH as it can be seen in figure S9, right.

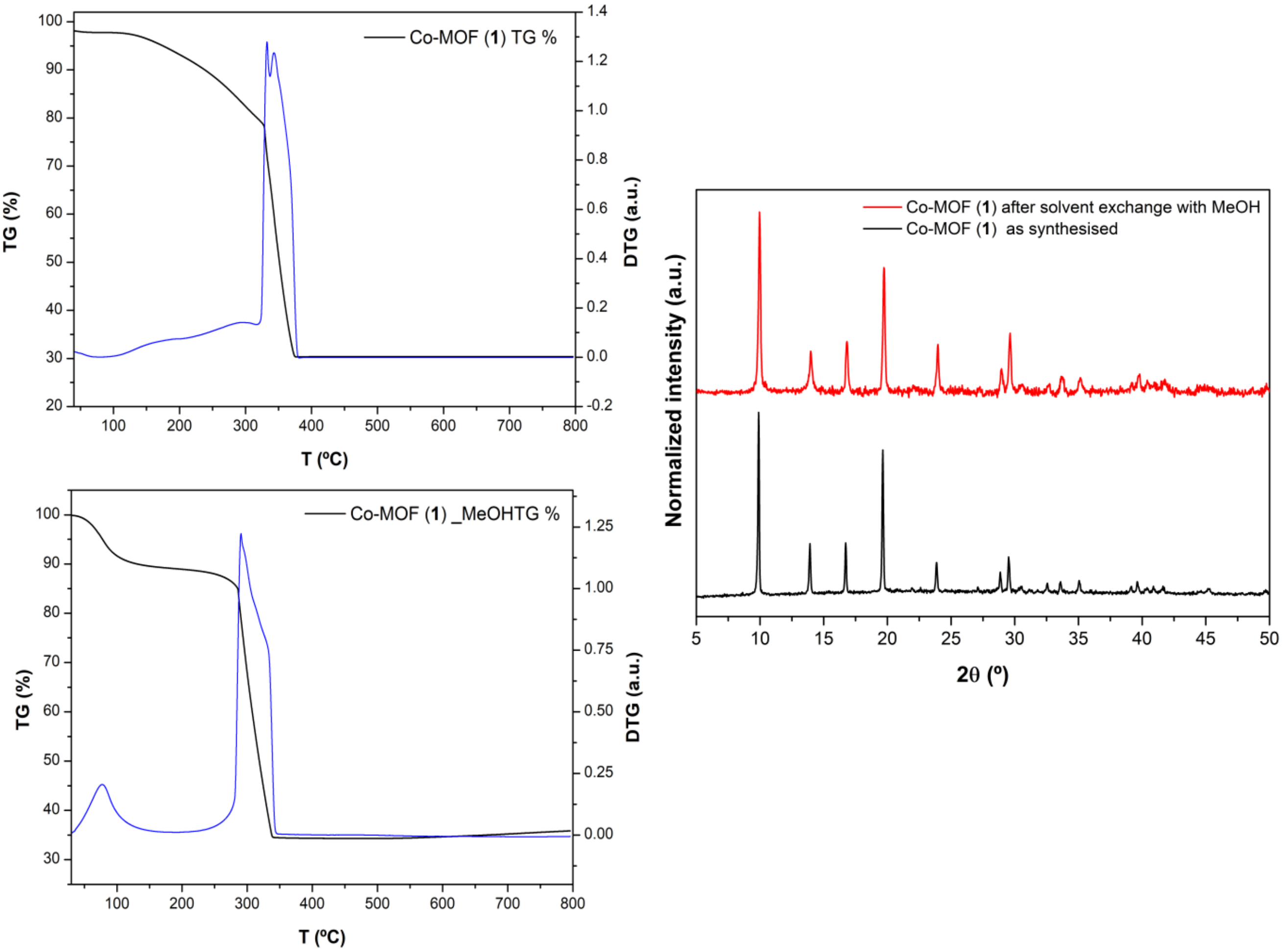

Figure S5. Figure of TG/DTA analysis of Co-MOF (left; up, as synthesised, down, after solvent exchange with MeOH) and figure of the experimental PXRD for Co-MOF before and after solvent exchange with MeOH (right).

TG curve of Co-MOF shows two main steps of weight loses. The first step concerns to the progressive loss of the solvent lattice molecules (DMF and H_2O) which are released from room

temperature up to 300ºC. Then, an abrupt descent can be seen that corresponds to the collapse of the crystal structure. From the shape of the TG curve, it seems that solvent molecules stabilise the structure and their removal promote crystal structure decomposition.

However, TG curve of Co-MOF after solvent exchange with methanol shows two well defined steps. The first step comprises the loss of lattice solvent molecules, which occurs at 100 ºC, and agrees with an efficient DMF to MeOH exchange. Subsequently, from 110 ºC to 300 ºC the TG curve of the compound describes a plateau, where the empty skeleton of the MOF is gotten. Finally, at 300 ºC ligand decomposition occurs and involves the collapse of the crystal structure, evolving to Co_3O_4 that is obtained at 800 ºC as the final residue.

8. Thermal evolution

Thermal evolution of Co-MOF shows that the compound maintains its crystallinity up to 300 ºC. These results come in line with thermogravimetric analysis, where it could be seen that above this temperature structure collapses and evolves into the metallic residue Co_3O_4 at around 800 ºC.

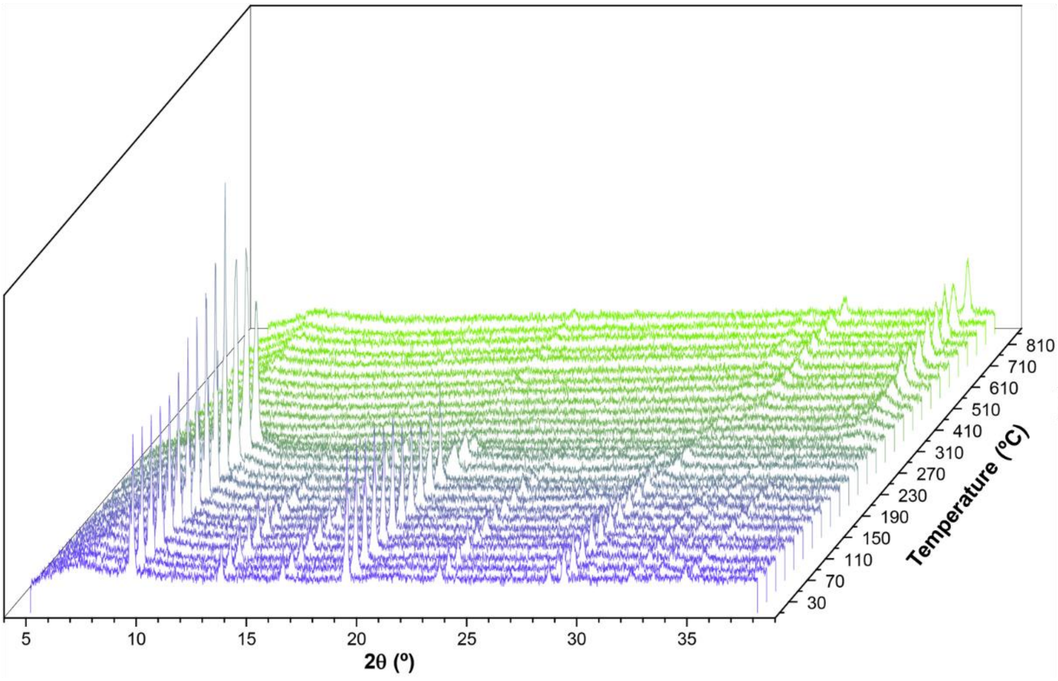

Figure S6. Thermal evolution of Co-MOF**.**

9. Additional views of the structure

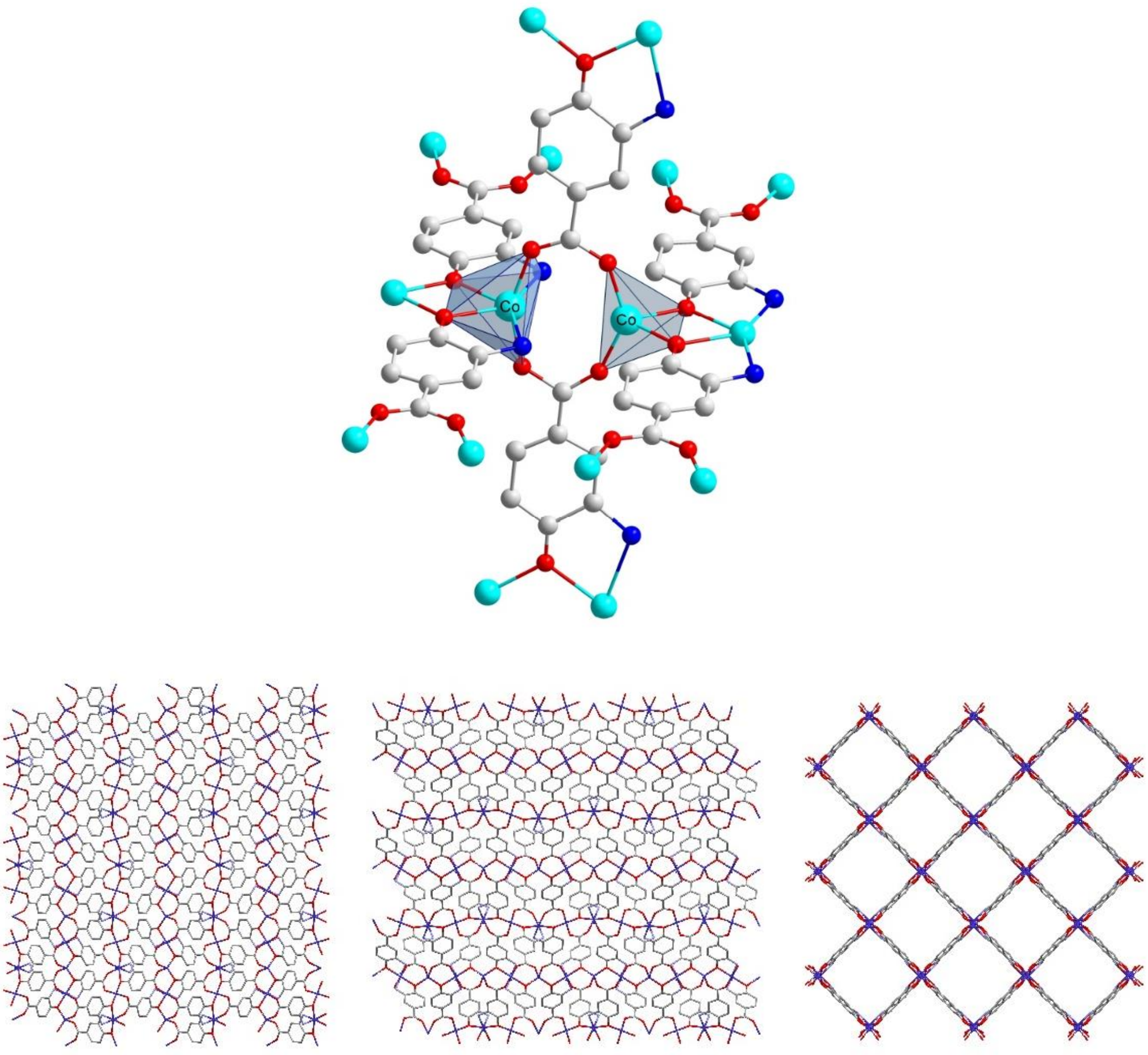

Co-MOF

Figure S7. View along a (left), b (middle) and c (right) axis of Co-MOF (down).

10.*Ac* magnetic susceptibility measurements

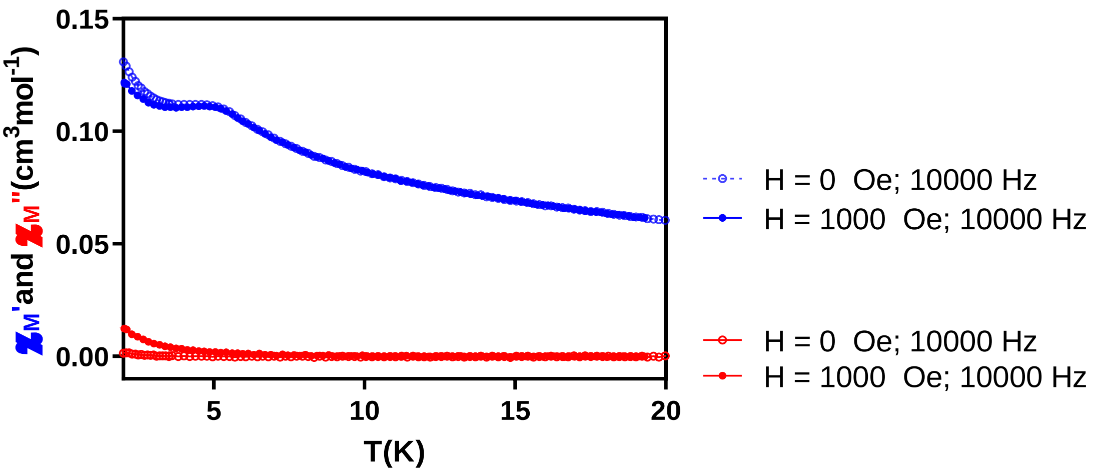

Figure S8. Temperature dependence of in-phase (blue) and out of phase (red) components of the *ac* susceptibility in a *dc* applied field of 1000 Oe for Co-MOF.

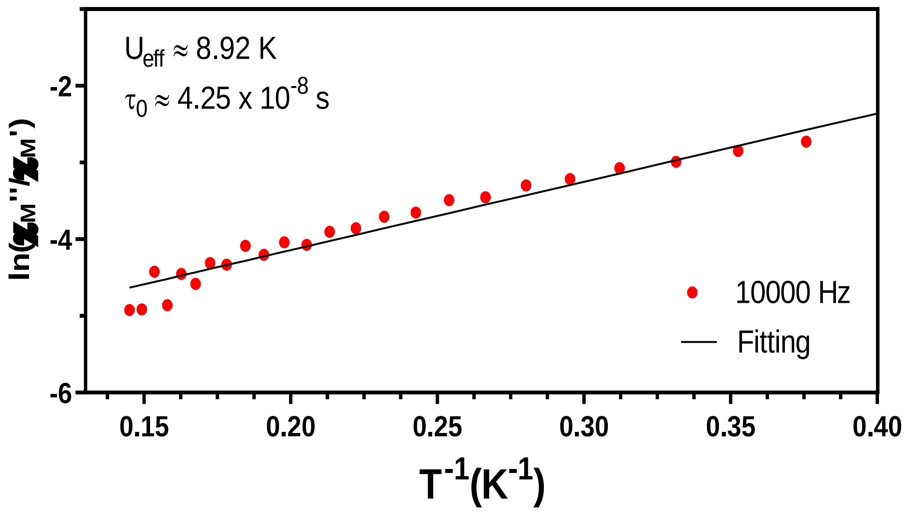

Figure S9. Plot of $\ln(\chi_M''/\chi_M')$ versus 1/T at 10000 Hz for Co-MOF under an applied field of 1000 Oe. The solid lines represent the linear fit with $\ln(\chi_M''/\chi_M') = \ln(2\pi\nu\tau_0) + E_a/k_BT$.

Table S6. Calculated orbital geometry related parameters.

Parameters	TPR	Td
D ($D_{KD1–2}$, $D_{KD1–3}$)/ cm^{-1}	-58.7 (-48.6, 46.7)	-31.4 (25.2, -28.1)
E/*D*	0.21	0.18
g_{xx}, g_{yy}, g_{zz}	2.08, 2.45, 2.93	2.16, 2.28, 2.6
Δ*E*(1–2), Δ*E*(1–3) / cm^{-1}	2367.2, 5325.4	3432.7, 4453.7

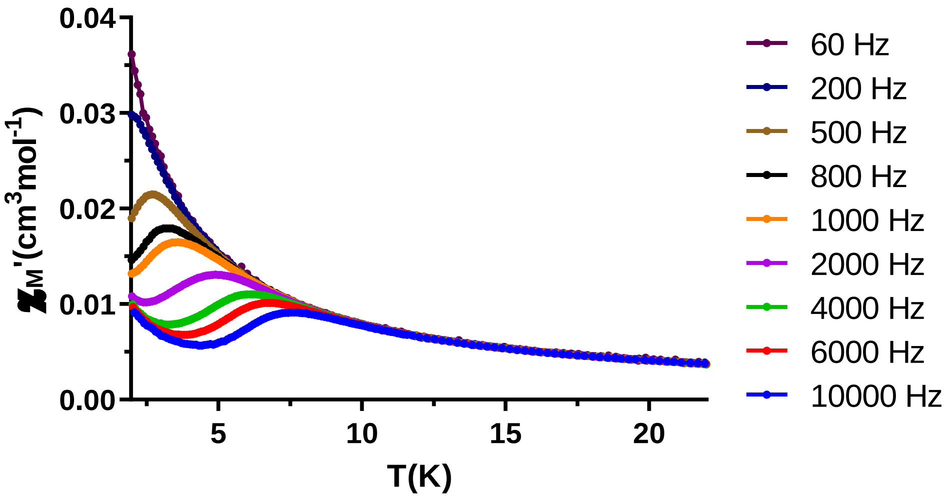

Figure S10. Temperature dependence of in-phase components of the *ac* susceptibility in a *dc* applied field of 1000 Oe for $[Co_{0.05}Zn_{0.95}L]_n$

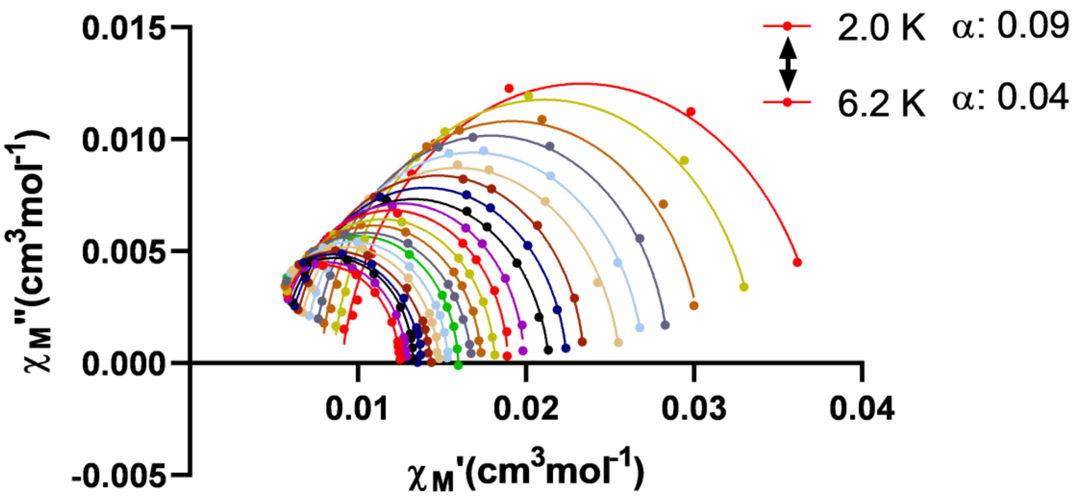

Figure S11. Cole-Cole plots in a *dc* applied field of 1000 Oe for $[Co_{0.05}Zn_{0.95}L]_n$

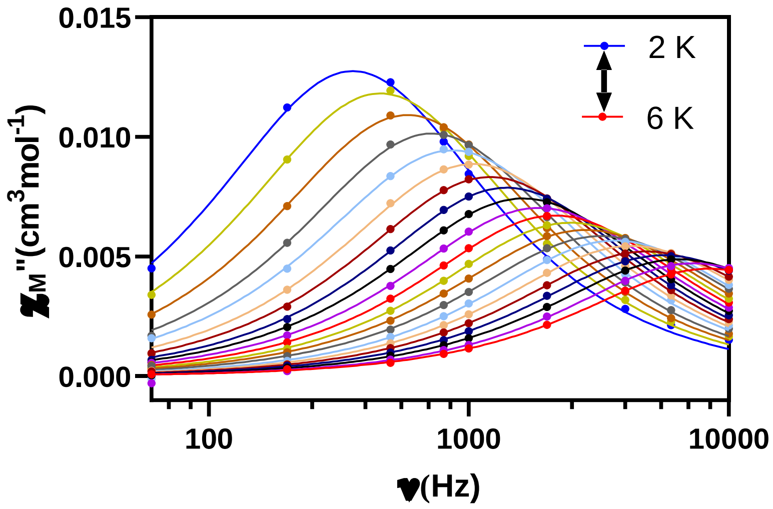

Figure S12. Variable-temperature frequency dependence of the χ_M'' signal under 1000 Oe applied field for $[Co_{0.05}Zn_{0.95}L]_n$. Solid lines represent the best fitting of the experimental data to the Debye model.

11.Diffuse reflectance measurements

Absorption spectrum of 3-amino-4-hydroxybenzoic acid ligand, pure Co^{II} and Zn^{II} homometallic compounds and $[Co_{0.05}Zn_{0.95}L]_n$ heterometallic compound show absorption bands in the range of 220–800 nm. Three main regions can be differentiated, the first one from 220–300 nm attributed to the ligand 3-amino-4-hydroxybenzoic acid ligand π–π* transitions, the shoulder at around 310 nm (clearly observed for both homometallic and heterometallic compounds) corresponds to metal-to-ligand charge transfer (MLCT) transitions. Moreover, the bands with a broad and structured shape covering the 400–700 nm region, are attributed to spin-allowed d-d transitions found in heterometallic $[Co_{0.05}Zn_{0.95}L]_n$ and homometallic Co^{II} compounds.

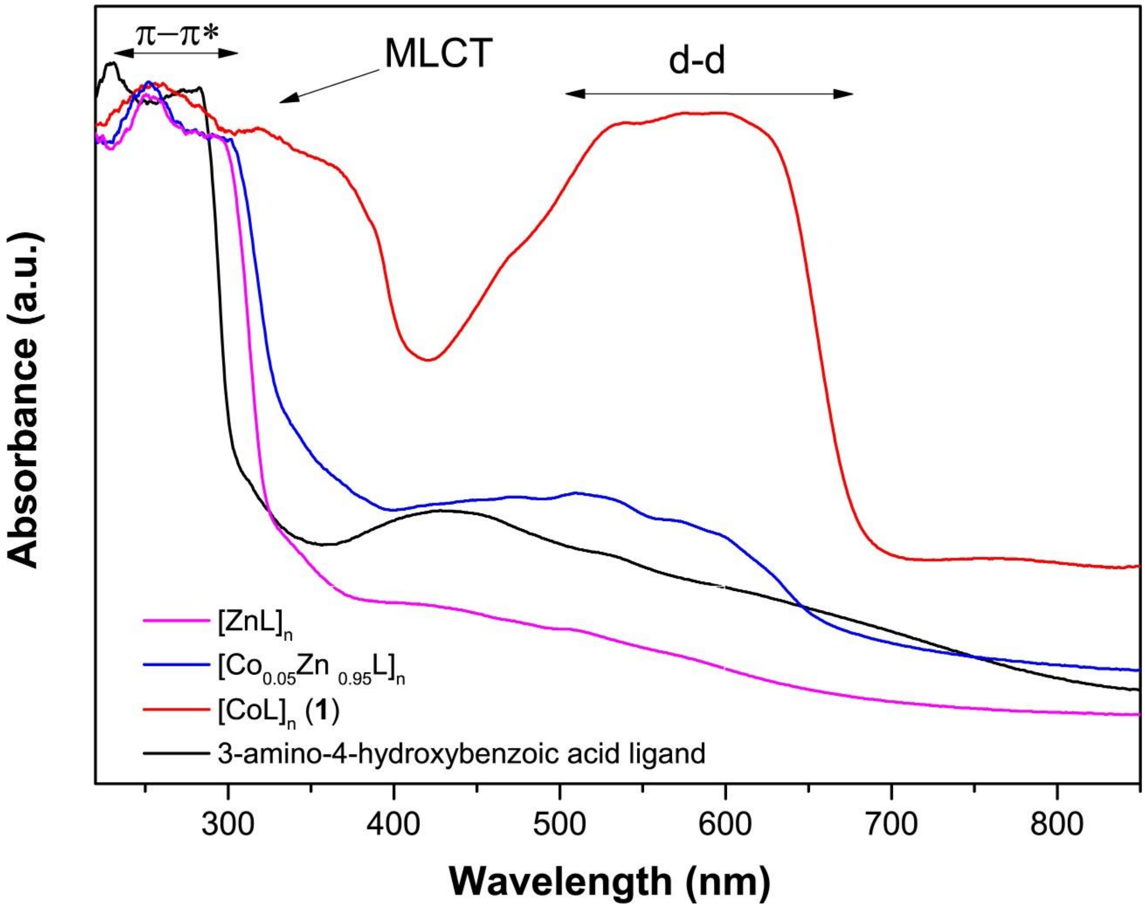

Figure S13. Diffuse reflectance of 3-amino-4-hydroxybenzoic acid ligand, homometallic Co-MOF, and Zn compounds and heterometallic $[Co_{0.05}Zn_{0.95}L]_n$ heterometallic sample.

12. Photoluminescence properties

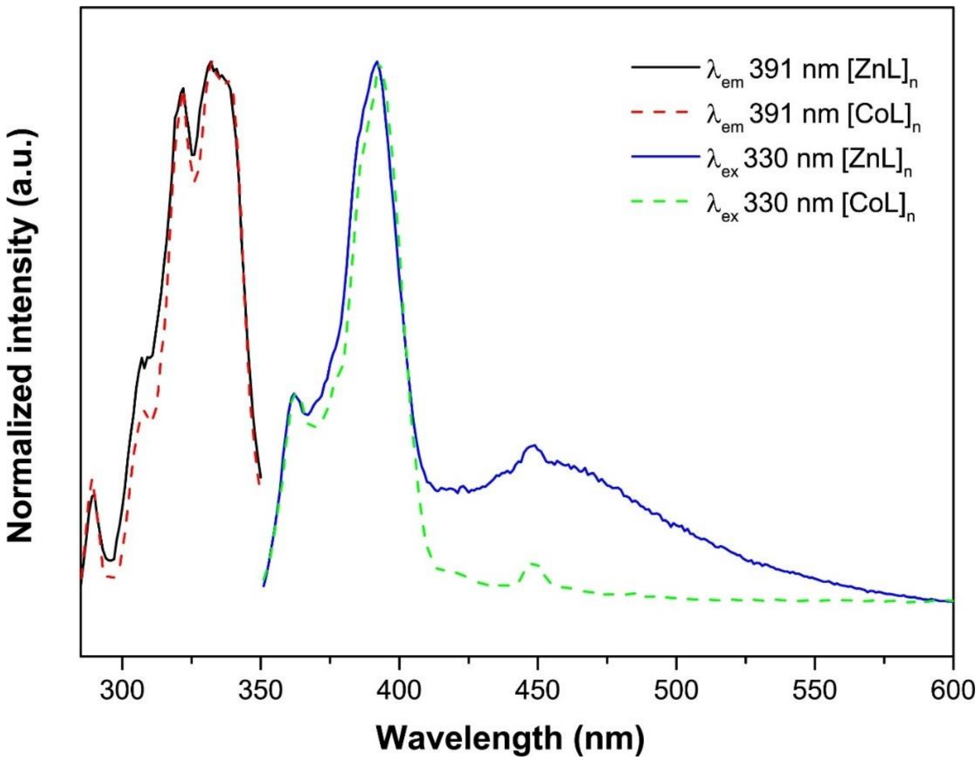

Figure S14. Figure caption of the experimental room temperature photoluminescence excitation and emission spectra under λ_{em} = 391 nm and λ_{ex} = 330 nm, respectively for compounds **1** and isostructural Zn^{II} homometallic counterpart.

The normalized excitation and emission spectra of Co-MOF and isostructural $[ZnL]_n$ compounds are sown in Figure S14.

The excitation spectra have been measured for both homometallic compounds by monitoring the emission maxima, at 391 nm. As observed in Figure S14, the excitation spectra show a band covering the 275–350 nm range in which four peaks (sited at ca. 288, 310, 322 and 334 nm) are distinguished.

Ambient temperature emission spectra monitored at the excitation maxima, at 330 nm, show a main band with the maxima at 362 nm and 391 nm and a tail. In case of homometallic Zn^{II} compound a more prominent shoulder can be appreciable peaking at 447 nm.

Normalization of the spectra has been carried out in order to compare the position of the maxima in both structures more than to compare the relative intensity, which in case of Co^{II} was, as expected, relatively weaker.
